# A43 SEX DIFFERENCES IN THE ASSOCIATION OF HIV WITH METABOLIC DYSFUNCTION-ASSOCIATED FATTY LIVER DISEASE (MAFLD)

**DOI:** 10.1093/jcag/gwac036.043

**Published:** 2023-03-07

**Authors:** D Kablawi, T F Tadjo, J Milic, W Elgretli, C Gioè, B Lebouché, A Cascio, G Guraldo, G Mazzola, G Sebastiani

**Affiliations:** 1 Department of Medicine, McGill University, Montreal , Canada; 2 Modena HIV Metabolic Clinic, University of Modena and Reggio Emilia, Modena, Italy; 3 Division of Experimental Medicine, McGill University, Montreal , Canada; 4 Department of Health Promotion Sciences and Mother and Child Care “Giuseppe D’Alessandro”, , University of Palermo, Palermo, Italy; 5 Department of Family Medicine, McGill University, Montreal, Canada

## Abstract

**Background:**

People with HIV (PWH) are at high risk for metabolic dysfunction-associated fatty liver disease (MAFLD). In the general population, sex differences seem to exist in frequency and severity of MAFLD, with higher prevalence of MAFLD in men, but higher incidence of liver fibrosis in women. Less is known about sex differences in MAFLD and liver fibrosis in the setting of HIV infection.

**Purpose:**

The aim of this study is to investigate the sex differences in the prevalence of MAFLD in PWH and the severity of liver fibrosis among this population.

**Method:**

This was a multicenter cohort study including consecutive PWH who underwent screening for MAFLD and liver fibrosis by liver stiffness measurement (LSM) with associated controlled attenuation parameter (CAP). MAFLD was defined as the presence of hepatic steatosis, diagnosed as CAP>270 dB/m, plus any among type 2 diabetes, overweight (BMI>25 Kg/m^2^) or two other metabolic abnormalities. Significant liver fibrosis was diagnosed as LSM>8 kPa. Incidence of MAFLD and significant liver fibrosis was assessed through survival analysis.

**Result(s):**

1359 PWH (25% females, 30% HCV coinfected) were included. Prevalence of MAFLD at baseline was lower in women than in men with HIV (17.7% vs. 24.3%, p=0.013). Conversely, there was no difference in prevalence of liver fibrosis (10.7% vs. 13.4%). Women with MAFLD were more frequently of black ethnicity (48% vs. 14%, p<0.001), had lower ALT (26.4+20.4 vs. 33.4+22.5; p=0.035), higher HDL cholesterol (1.46+0.57 vs. 1.11+0.33; p<0.001), lower triglycerides (1.69+0.96 vs. 2.47+2.63; p=0.035) compared to men with MAFLD. 485 of these PWH were followed for a median of 3.5 years. Incidence of MAFLD was similar between women and men with HIV. However, incidence of liver fibrosis was higher in women compared to men with HIV (7.0 per 100 person-years [PY] vs. 5.9 per 100 PY; p=0.035) (Figure 1). The higher incidence of significant liver fibrosis occurred particularly after the age of 50 years. On multivariable Cox regression analysis and after adjusting for age, presence of MAFLD (adjusted hazard ratio [aHR] 3.3, 95% CI 2.0-5.6) and female sex (aHR 2.2, 95% CI 1.3-3.5) were independent predictors of developing significant liver fibrosis while CD4 cell count was protective (aHR 0.99, 95% CI 0.99-0.99).

**Image:**

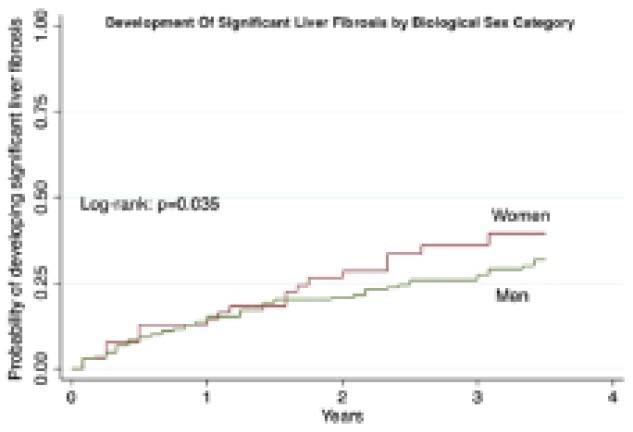

**Conclusion(s):**

MAFLD seems a sexual dimorphic disease in PWH. Despite having lower rates of MAFLD, women with HIV have higher incidence of significant liver fibrosis compared to men, especially after 50 years of age. Future studies should target adequate consideration of sex differences in clinical investigation of MAFLD to fill current gaps and implement precision medicine for PWH.

**Please acknowledge all funding agencies by checking the applicable boxes below:**

None

**Disclosure of Interest:**

None Declared

